# Moderate evidence exists for four microRNAs as potential biomarkers for tendinopathies and degenerative tendon ruptures at the upper extremity in elderly patients: conclusion of a systematic review with best-evidence synthesis

**DOI:** 10.1186/s40634-023-00645-5

**Published:** 2023-08-10

**Authors:** Tristan Schmid, Florian Wegener, Thilo Hotfiel, Matthias W. Hoppe

**Affiliations:** 1 Movement and Training Science, Leipzig University, Jahnallee 59, 04109 Leipzig, Germany; 2 Center for Musculoskeletal Surgery Osnabrück (OZMC), Klinikum Osnabrück, Am Finkenhügel 1, 49076 Osnabrueck, Germany

**Keywords:** Circulating RNA, Ci-miRNA, Micro-RNA, miRNA, Connective tissue, Tendinopathy, Tendinitis, Tear, Tendon pathology, Shoulder pathology, Overuse

## Abstract

**Purpose:**

The aim of this systematic review was to investigate tendon-specific microRNAs (miRNAs) as biomarkers for the detection of tendinopathies or degenerative tendon ruptures. Also, their regulatory mechanisms within the tendon pathophysiology were summarized.

**Methods:**

A systematic literature research was performed using the PRISMA guidelines. The search was conducted in the Pubmed database. The SIGN checklist was used to assess the study quality of the included original studies. To determine the evidence and direction of the miRNA expression rates, a best-evidence synthesis was carried out, whereby only studies with at least a borderline methodological quality were considered for validity purposes.

**Results:**

Three thousand three hundred seventy studies were reviewed from which 22 fulfilled the inclusion criteria. Moderate evidence was found for miR-140-3p and miR-425-5p as potential biomarkers for tendinopathies as well as for miR-25-3p, miR-29a-3p, miR-140-3p, and miR-425-5p for the detection of degenerative tendon ruptures. This evidence applies to tendons at the upper extremity in elderly patients. All miRNAs were associated with inflammatory cytokines as interleukin-6 or interleukin-1ß and tumor necrosis factor alpha.

**Conclusions:**

Moderate evidence exists for four miRNAs as potential biomarkers for tendinopathies and degenerative tendon ruptures at the upper extremity in elderly patients. The identified miRNAs are associated with inflammatory processes.

## Introduction

Tendons are a key element in the musculoskeletal system for the generation of movements due to their ability to transmit and withstand forces [1]. However, pathological tendon conditions such as tendinopathies are prevalent in the entire population with incidences of up to 10.52 per 1,000 persons per year [2]. Tendinopathies are characterized by persistent tendon pain and loss of function associated with mechanical loading [3] and could cause a reduced life quality [4], impairments of work and sportive performances [5], and underestimated high socio-economic costs [6]. The pathogenesis is understood as a continuum model with the end stage of degenerative tendinopathy [7], where symptoms may persist for decades [8]. Since associated degenerative changes are present in 97% of all ruptured tendons [9], it is assumed that tendinopathies can cause such acute severe tendon injuries [10]. However, high-quality evidence for effective preventive measures for tendinopathies is lacking [11, 12] and early clinical management is challenging due to asymptomatic early stages [13] as well as often ignored minor symptoms [14]. In this context, established clinical routine diagnostics such as anamnesis, clinical examination, and tendon imaging [15] are suitable for the diagnosis of manifested tendinopathies, but inappropriate for asymptomatic early stages. Thus, more research is needed to evaluate diagnostic tools for the early diagnosis of tendinopathies and associated degenerative tendon ruptures, including the identification of potential biomarkers.

MicroRNAs (MiRNAs) are short noncoding RNA molecules that bind to complementary messenger-RNAs to regulate their activity [16]. In humans, miRNAs are expressed in a cell- and tissue-specific manner [17, 18]. They can be detected in a variety of different body fluids including blood, tears, or saliva [19]. MiRNAs are suitable diagnostic biomarkers [20], because they are protected from endogenous RNAse activity [21] and can endure freeze–thaw cycles [22]. In this context, miRNAs have been evaluated as non- or minimal-invasive biomarkers for numerous diseases including Alzheimer [23], multiple sclerosis [24], heart failure [25], or various cancer types [26–28], but little is known with respect to degenerative tendon conditions yet.

MiRNAs have been associated with the tendon tissue pathophysiology. It has been demonstrated that miRNAs could reduce adhesion, enhance remodeling, and promote angiogenesis in the context of tendon healing [29]. Also, miRNAs are known to regulate a variety of different genes related to tendon healing and tenogenesis [30]. To date, there are two systematic reviews investigating the relationship between the expression rates of miRNAs and tendon tissue functions. Dubin et al. [31] investigated the effect of miRNAs on tenocytes and tendon-related gene expression. They show that miRNAs have both positive and negative effects on the tendon tissue homeostasis. Giordano et al. [32] examined the therapeutic potential of miRNAs in the context of tendon healing. The authors conclude that miRNAs could serve as useful therapeutic targets due to their influence on the expression of cytokines and differentiation and proliferation of stromal cell lines involved in the composition of the extracellular matrix. However, there is no systematic review questioning, if miRNAs can be used as biomarkers for pathological tendon conditions. Therefore, the aim of this systematic review was to investigate tendon-specific miRNAs as biomarkers for the detection of tendinopathies or degenerative tendon ruptures. Also, the regulatory mechanisms of miRNAs within the tendon pathophysiology were summarized.

## Methods

### Research design

The systematic review was conducted using the Preferred Reporting Items for Systematic review and Meta-Analysis Protocols (PRISMA) [33]. The inclusion and exclusion criteria were determined using a PICO(S) scheme: i.e., population (P), intervention (I), comparison (C) outcome (O), and study design (S) [34]. Additionally, the item "other" was included to account for further criteria (Table 1). The inclusion criteria were: (i) human studies including patients with tendinopathies or degenerative tendon ruptures; (ii) tendon-specific miRNAs quantified in the tissue and/or circulation; (iii) primary data published in original investigations; (iv) publication language in English or German; and (v) full text availability. Studies were excluded, when the miRNAs were not specified. All methodological steps were conducted by one author and a second validated them. In terms of uncertainties, it was discussed until a consensus was reached. Due to the non-invasive character, no ethical approval was considered.

**Table 1:** PICO(S) scheme for the definition of the inclusion and exclusion criteria

	**Population**	**Intervention**	**Comparison**	**Outcome**	**Study Design**	**Other**
Inclusion criteria	Human studies: subjects of any gender and any age	Measurement of miRNAs associated with tendons, tendinopathies, tendinitis, tendosynovitis, tendinosis, or tenocytes Measurement of miRNAs from body fluids or by sample collection from biopsies	Intrapersonal comparisons, interpersonal comparisons, pre-post comparisons at one or more time points	Results provide information about tendon-specific miRNAs associated with tendinopathies or degenerative tendon ruptures Information about regulatory mechanisms or expression patterns of tendon-specific miRNAs	Original data within interventional and descriptive studies	English or German language Studies with full access
Exclusion criteria				No information about specific miRNAs available		

*PICO(S)* Population Intervention Comparison Outcome Study design, *miRNA* microRNA

### Literature search strategy and study selection

The search was performed in the meta-database Pubmed on 04/25/2022 and was not restricted to a specific time period. To find relevant studies, a search line was elaborated using the inclusion and exclusion criteria. The search line included the following terms: (micro RNA OR miR OR miRNA OR microRNA OR circRNA OR circulating RNA OR ciRNA) AND (tendon OR tendinopathy OR tendinosis OR tendinitis OR tendosynovitis OR tenocytes OR ruptures OR connective tissue) AND (physiology OR pathology OR pathophysiology OR maladaptation OR load OR intervention OR adaptation OR baseline OR timepoint OR pre-post OR comparison). Additionally, the reference list of two previous systematic [31, 32] and five previous narrative reviews [29, 30, 35–37] within the particular research field were screened for further suitable studies. After duplicates were removed, the abstracts and full texts of the remaining studies were checked for their fit by taking the eligibility criteria into account.

### Risk of bias assessment

The study quality and associated risk of bias was determined using the Scottish Intercollegiate Guidelines Network (SIGN) checklist [38]. Therefore, the particular checklist for randomized controlled trials, cohort studies, case–control studies, and diagnostic and economic studies was used. The checklists consisted of 10–15 items to test the internal validity of the studies. The items were rated as "Yes" (Y), "No" (N), "Can't say" (CS), or "not applicable" (NA). The overall rating of the studies involved the following outcomes: "high quality", "acceptable quality", "borderline quality", or "unacceptable quality", as described in detail elsewhere [39].

### Data extraction

The data extraction of the studies was conducted according to the PICO(S) scheme. For validity, studies with an unacceptable quality were not considered, as conducted previously [39]. Due to the found heterogeneity in terms of the methodologies and results of the studies, no meta-analysis was performed. Instead, a best-evidence synthesis was conducted to clarify the evidence and direction of the miRNA expression rates [39]. The expression rates and their associations with tendinopathies or degenerative ruptures were classified as: upregulated (↑), downregulated (↓), or neutral ( →), which means that no clear pattern was given. To increase the validity, only miRNAs that were found, at least in part, twice in different studies were considered in the best-evidence synthesis. An exception was made for the study by Thankam et al. [40], where only the 10 most up- and down-regulated miRNAs were included to reduce the amount of data from this comprehensive microarray study including more than 235 miRNAs. Nevertheless, miRNAs that occurred more than two times were matched to the study by Thankam et al. [40], if they were not already included in the 10 most up- or down-regulated in this study. Table 2 summarizes the applied criteria for the best-evidence synthesis according to Asker et al. [39], whereby the final ratings were as follows: “strong evidence”, “moderate evidence”, “limited evidence”, and “no evidence”.

**Table 2:** Criteria for the best-evidence synthesis according to Asker et al. [39]

Rating	Study quality	Criterion
Strong evidence	≥ 2 high quality studies	≥ 75% consistent findings in these studies
Moderate evidence	1 high quality studies and/or ≥ 2 moderate quality studies	≥ 75% consistent findings in these studies
Limited evidence	1 moderate quality study and/or ≥ 1 low quality study	n/a
Conflicting evidence	≥ 2 studies of any quality	< 75% consistent findings in these studies
No evidence	No admissible studies were found	

*n/a* not applicable

## Results

### Literature search strategy, study selection, and risk of bias

**Fig. 1:**
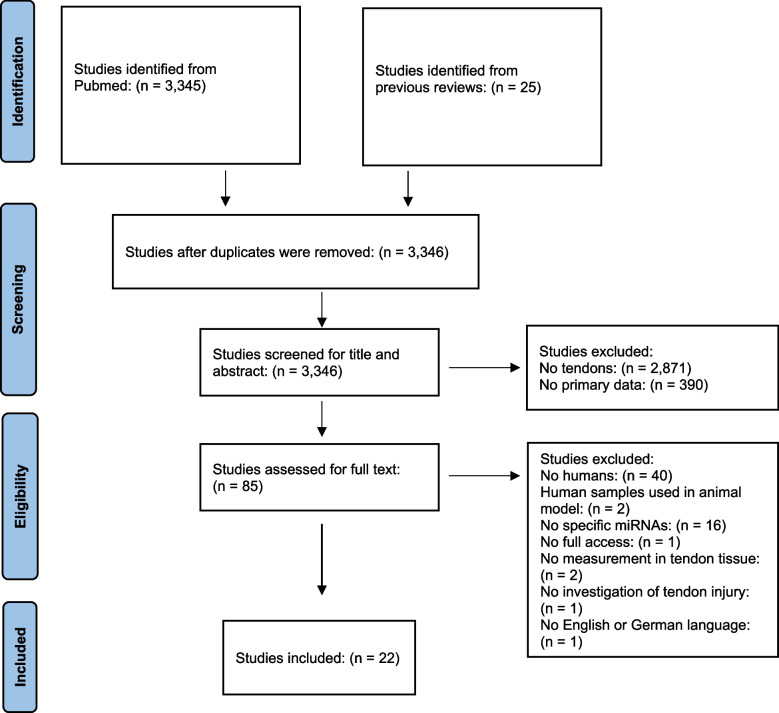
Flow chart of the literature search strategy according to the PRISMA guidelines

Figure 1 shows the results of the literature search strategy and study selection. 3,345 and 25 articles were found using the search line and reference lists, respectively. After duplicates were removed, 3,346 articles remained. Thereof, 3,324 articles were excluded due to different reasons (Fig. 1). Thus, a total of 22 studies were finally included and considered for the risk of bias assessment.

Table 3 shows the results of the risk of bias assessment by the SIGN-checklist. Of the 22 considered studies, one study was classified as high quality [41], three studies as acceptable [42–44], 14 as borderline [40, 45–57], and four as unacceptable [58–61].

**Table 3:** Results of the 22 studies checked for the risk of bias assessment using the SIGN checklist

Study	Item											Total				Overall Assessment
	**1.1**	**1.2**	**1.3**	**1.4**	**1.5**	**1.6**	**1.7**	**1.8**	**1.9**	**1.10**	**1.11**	**Y**	**N**	**CS**	**NA**	
Plachel et al. [41]	Y	Y	Y	100	Y	Y	Y	CS	Y	Y	Y	9	0	1	0	High quality
Ge et al. [42]	Y	Y	Y	100	N	Y	Y	CS	Y	N	Y	7	2	1	0	Acceptable
Hall et al. [43]	Y	Y	Y	100	Y	Y	Y	CS	Y	N	N	7	2	1	0	Acceptable
Leal et al. [44]	Y	Y	Y	100	Y	Y	Y	CS	Y	N	Y	8	1	1	0	Acceptable
Feng et al. [47]	Y	N	N	N	Y	Y	Y	0	CS	Y	-	5	3	1	0	Borderline
Sun et al. [53]	Y	N	N	N	Y	N	Y	0	CS	Y	-	5	3	1	0	Borderline
Xiao et al. [57]	Y	N	N	N	Y	CS	Y	0	CS	Y	-	4	3	2	0	Borderline
Thankam et al. (2019)	Y	CS	CS	100	N	Y	Y	CS	Y	N	Y	5	2	3	0	Borderline
Ge et al. [48]	Y	Y	Y	100	N	Y	Y	CS	Y	N	N	6	2	2	0	Borderline
Thankam et al. [40]	Y	CS	CS	100	N	Y	Y	CS	Y	N	N	4	3	3	0	Borderline
Han et al. [49]	Y	Y	CS	100	N	Y	Y	CS	Y	N	Y	6	2	2	0	Borderline
Brown et al. [46]	Y	CS	Y	91	CS	Y	Y	CS	Y	N	N	5	2	3	0	Borderline
Lu et al. [50]	Y	N	N	N	CS	CS	Y	0	CS	Y	-	3	3	3	0	Borderline
Thankam et al. [54]	Y	CS	CS	100	N	Y	Y	CS	Y	N	Y	5	2	3	0	Borderline
Wang et al. [56]	Y	N	N	N	Y	Y	Y	0	CS	Y	-	5	3	1	0	Borderline
Millar et al. [51]	Y	N	N	N	Y	CS	Y	0	CS	Y	-	4	3	2	0	Borderline
Peffers et al. [52]	Y	Y	CS	100	N	Y	Y	CS	Y	N	N	5	3	2	0	Borderline
Abrahams et al. [45]	Y	Y	CS	100	CS	Y	Y	CS	Y	N	N	5	2	3	0	Borderline
Hu et al. [60]	Y	N	N	N	CS	CS	Y	CS	CS	CS	-	2	3	5	0	Unacceptable
Chen et al. [59]	Y	N	N	N	CS	CS	Y	CS	CS	CS	-	2	3	5	0	Unacceptable
Cai et al. [58]	Y	CS	CS	100	CS	Y	Y	NA	Y	N	N	4	2	3	1	Unacceptable
Poulsen et al. [61]	Y	N	N	N	CS	CS	Y	CS	CS	CS	-	2	3	5	0	Unacceptable

*SIGN* Scottish Intercollegiate Guidelines Network, *Y* Yes, *N* No, *NA* Not applicable, *CS* Can’t say

### Study characteristics

Table 4 summarizes the study characteristics of the 22 studies according to the PICO(S) scheme. Concerning the study design, there were 13 case–control [40–46, 48, 49, 52, 54, 55, 58] and 9 controlled studies [47, 50, 51, 53, 56, 57, 59–61]. In total, miRNAs were quantified for 15 times in the tissue [40, 42–44, 48, 49, 51–55, 57, 59–61] and two times in the circulation [46, 47]. Two studies considered both [41, 50] and in three studies the sample was unclear [45, 56, 58]. With respect to the tissue, the biopsy was taken four times from the supraspinatus tendon [42, 44, 48, 53], three times from the bicep tendon [40, 54, 55], three times from the Achilles tendon [49, 52, 59], twice from both the supraspinatus and subscapularis tendons [43, 51], twice from patellar tendon [57, 60], and once from the hamstring tendon [61]. In regard to the circulation, miRNAs were detected in one study each from whole blood [47] and saliva [46]. In the study in which the samples were taken from both the tissue and circulation, measurements were taken from venous blood as well as from the supraspinatus and subscapularis tendons [41]. In another study, mesenchymal stem cells were harvested from bone marrow and tendon stem cells from hamstring tendon and the effect of miR-29b-3p on the expression of transforming growth factor ß1 (TGF-ß1) and type I collagen was tested [50]. To quantify miRNA expression rates, 17 studies used PCR methodology [41, 42, 44–53, 56, 57, 59–61], four studies performed microarray analysis [40, 54, 55, 58], and one study used RNA PICO quantitation method [43]. Concerning the microarray approaches, three studies used biceps tendon samples [40, 54, 55], whereas the sample was unclear in one study [58]. Table 5 summarizes the regulatory mechanisms of the miRNAs of the included 22 studies.

**Table 4:** Characteristics of the included studies according to the PICO(S) scheme

Author	Population	Intervention	Localization	Outcome
Feng et al. [47]	6 healthy male subjects, 31 ± 4 years of age	An experimental group was treated with miR-6924-5p and compared with a comparison group (no treatment) for various osteoclastogenesis markers	Whole blood (monocytes)	Monocytes treated with the miR-6924-5p had significantly downregulated osteoclastogenesis markers compared to the control group
Sun et al. [53]	10 male and 16 female patients, 60 ± 7 years of age	An in vitro model was used to investigate the function of HMGA2 in human tendon stem cells treated with H2O2	Supraspinatus tendon	H2O2 leads to increased Nudt21 expression and thus increased let-7 production in tendon stem cells
Ge et al. [42]	2 male and 8 female subjects 5 patients 47–71 years of age and 5 subjects 44–66 years of age	Profiling of lncRNAs, mRNAs and miRNAs involved in rotator cuff tendinopathy in comparison with healthy tendons	Supraspinatus tendon	The analysis identified 35 miRNAs whose expression was significantly altered in tendinopathies compared with healthy tendons
Plachel et al. [41]	Investigation 1: 2 male and 3 female healthy subjects, 58.1 ± 6 years of age, 3 male and 2 female patients, 57.0 ± 5.9 years of age, 2 male and 2 female patients, 60.1 ± 8.4 years of age Investigation 2: 1 male and 3 female patients 62.4 ± 10.1 years of age, 5 male and 2 female patients, 64.8 ± 7.9 years of age,8 healthy male subjects, 29.8 ± 8.1 years of age	miRNA profiles were compared between healthy subjects as well as patients with chronic tendinopathies and patients with degenerative rotator cuff tears	Venous blood, Subscapularis tendon, Supraspinatus tendon	Several miRNAs were found to be significantly dysregulated when comparing the different groups
Xiao et al. [57]	2 healthy subjects, sex unclear, mean age 24.5 years	Human tenocytes were treated with miR mimics and antagomirs of miR-30d, 26a, and 29a. Subsequently, gene expression was evaluated for scleraxis, collagen 1 alpha 1, collagen 3 alpha 1, IL-1β, IL-6, BMP2, BMP12, and osteocalcin	Patellar tendon	miR-29a mimics and mir-29a-antagomir resulted in a significant reduction of BMP2 in human tenocytes. In addition, there was a significant reduction of BMP12 by miR-29a mimics
Thankam et al. [55]	8 patients, sex and age unknown	Tendon samples were compared between one group with tendon injury and fat infiltration vs. one group with tendon injury but without fat infiltration to find out which miRNAs are different	Biceps tendon	13 highly significant miRNAs and 216 target genes were identified
Hall et al. [43]	5 male patients, 44–65 years of age	One tendinopathic supraspinatus tendon and one healthy subscapularis tendon from each of 5 patients were biopsied, and the expression of miRNAs was compared	Subscapularis tendon, Supraspinatus tendon	Twenty-one miRNAs were identified that showed significantly altered expression between the healthy and tendinopathic tendons
Ge et al. [48]	Patients, n unknown, sex unknown, age 40.4 ± 10.3 years of age and patients, n unknown, sex unknown, 36.3 ± 11.5 years of age	Investigation of the role of miR-148a-3p in the development of angiogenesis in tendinopathies	Supraspinatus tendon	The miR-148a-3p is significantly upregulated in tendinopathic tendons. miR-148a-3p upregulates the expression of thrombospondin-4 and promotes angiogenesis by inhibiting Krüppel-like factor 6
Thankam et al. [40]	8 patients, sex and age unknown	Investigation of miRNAs associated with the JAK2/STAT3 pathway. In addition, target genes associated with glenohumeral arthritis and rotator cuff tears were identified	Biceps tendon	235 miRNAs were identified whose expression was significantly altered between group 1 and group 2. In addition, 284 target genes related to the JAK/STAT3 pathway were identified
Han et al. [49]	Young healthy subjects, n unknown, sex unknown, 25 ± 8 years of age, old subjects with tendon degeneration, n unknown, sex unknown, age 65 ± 10 years	It was investigated whether the senescence marker p16 affects age-related tenogenic differentiation in tendon stem/progenitor cells (TSPCs). For this purpose, young and old TSPCs were compared. In addition, a mir-217 mimic or a miR-217 inhibitor was added to the TSPCs and the effect was examined	Achilles tendon	The miR-217 was significantly upregulated in old tendon stem cells, furthermore, an increase in p16 was detected with a parallel decrease in type 1 collagen. Downregulation of miR-217 reversed the inhibitory effect of p16 on tenogenic differentiation of old TSPCs
Leal et al. [44]	19 male, 21 female patients, age 56.2 ± 11.1 years, 5 male and 6 female subjects, age 57.5 ± 14.1 years without tendon injury	Comparison between injured and healthy tendons, regarding mRNA expression, DNA methylation status, the MMP and TIMP genes, and miR-29 family expression	Supraspinatus tendon	miR-29a-3p, miR-29b-3p, miR-29b-5p correlated (inversely) significantly with MMP2, MMP9, and MMP 14; in addition, miR-29a-3p and miR-29b-5p correlated significantly with MMP1. No differences in miRNA-29 family expression between injured and healthy tendons
Brown et al. [46]	130 healthy subjects, sex and age unknown, 112 patients sex and age unknown	Comparison between patients with chronic Achilles tendinopathy and healthy control group regarding 8 different genes, including MIR608, which encodes mir-608	Saliva	Individuals with MIR 608 genotype encoding miR-608 had significantly lower risk of Achilles tendon injury
Lu et al. [50]	2 healthy male patients, 38 and 43 years of age	It was investigated whether long non-coding RNA (lncRNA) H19 affects tenogenesis of human tendon stem cells. In addition, the effect of miR-29b-3p-mimics and anti-miR-29b-3p on H19 was investigated	Bone marrow, Hamstring tendon	The lncRNA H19 increases TGF-ß expression and promotes tenogenic differentiation by inhibiting miR-29b-3p. miR-29b-3p inhibited the expression of TGF-ß and type I collagen
Thankam et al. [54]	8 patients, sex and age unknown	Microarray analysis to determine which miRNAs play a critical role in tendon tissue inflammation	Biceps tendon	7 miRNAs were found to have a significant change in expression pattern in inflamed tendons
Wang et al. [56]	Unclear	The effect of miR-124 on collagen formation in TDSCs was investigated	Unclear	miR-124 controls collagen formation in TGF-ß1-induced differentiation of tendon stem cells by significantly inhibiting egr1 expression
Hu et al. [60]	3 male patients, mean age 26.5 years	This study aimed to investigate the osteogenic effects induced by extracorporeal shock waves on TDSCs among others, and their underlying mechanisms	Patellar tendon	miR-138 was significantly downregulated in TDSCs by extracorporeal shock waves, resulting in increased osteogenic differentiation
Chen et al. [59]	2 subjects, sex unknown 28 and 31 years	To investigate the role of PIN-1 in the aging of TSPCs. In addition, the role of miR-140-5p in association with PIN-1 was studied	Achilles tendon	miR-140-5p has a significant effect on PIN-1 expression, which is associated with senescence in tendon stem cells
Millar et al. [51]	17 patients, sex unknown, mean age 54 years, 10 healthy subjects, sex unknown mean age 35 years	The role of IL-33 in association with miR-29a in early tendinopathies was analyzed	Supraspinatus tendon, Subscapularis tendon	Addition of IL33 significantly downregulated miR-29a. Downregulation of miR-29a significantly increased collagen-3 production. Addition of a miR-29a mimic significantly decreased collagen-3 production
Cai et al. [58]	23 healthy subjects, sex and age unknown, 23 patients, sex and age unknown	Tendinopathic samples were compared with healthy control samples, and their miRNA expression was investigated via microrray analysis	Unclear	During the analysis, 15 miRNAs were located that showed a significantly different expression pattern
Peffers et al. [52]	2 male and 3 female patients 69.4 ± 7.3 years of age, 4 male patients 19 ± 5.8 years of age, 4 young subjects sex unknown, 16.7 ± 2.8 years of age 4 old subjects, sex unknown 73.2 ± 6.5 years of age	Gene expression analysis was performed, and the results found were compared between old and young individuals. Subsequent validation by control group	Achilles tendon	A total of 325 elements were found including one miRNA, miR-1245A, whose expression differed significantly between young vs. old individuals
Poulsen et al. [61]	Unclear	Tendon cells were cultured in high or low glucose concentration and the miRNAs which had a significant changed expression were determined	Hamstring tendon	High glucose (oxidative stress) leads to significant upregulation of miR-28-5p which results in apoptosis of tenocytes
Abrahams et al. [45]	342 asymptomatic subjects, 160 patients with chronic Achilles tendon tendinopathy, sex and age unclear	This study aimed to compare the polymorphism of individuals with Achilles tendinopathy with a healthy control group	Unclear	The MIR608 gene encoding miR-608 may be associated with Achilles tendon tendinopathies

*miR* micro-RNA, *HMGA2* High-mobility group AT-hook 2, *H2O2* hydrogen peroxide, *Nudt21* Nudix Hydrolase 21, *lncRNA* long non-coding RNA, *mRNA* messenger-RNA, *miRNA* microRNA, *IL-1ß* Interleukin 1-ß, *IL6* Interleukin 6, *BMP* Bone morphogenetic protein, *n* number of participants, *TSPCs* tendon stem/progenitor cells, *DNA* deoxyribonucleic acid, *MMP* matrix metalloproteinase, TIMP tissue inhibitors of metalloproteinases, *TGF-ß* transforming growth factor ß, *TDSC* tendon stem cell, *egr1* early growth response protein 1*, PIN1* Peptidyl-prolyl isomerase, *IL33* Interleukin 33

**Table 5:** Overview of the regulatory mechanisms of the miRNAs of the included 22 studies

Author	miRNA	Expression patterns and mechanisms
Feng et al. [47]	miR-6924-5p	Increase in miR-6924-5p resulted in significant decrease in osteoclastogenic markers
Sun et al. [53]	let-7	Increase in miR-let-7 resulted in significantly reduced protection of hTDSCs
Ge et al. [42]	miR-6791-5p, miR-4632-5p, miR-4739, miR-1285-3p, miR-6803-5p, mir-6752-5p, miR-4763-3p, miR-3960, miR-6089, miR-4459, miR-1915-3p, miR-6775-5p, miR-2861, miR-328-5p, miR-4685-5p, miR-1237-5p, miR-1273 g-3p, miR-6511b-3p, miR-6765-5p, miR-6722-3p, miR-939-5p, miR-6756-5p, miR-6724, miR-5787, miR-6889-5p, miR-6763-3p, miR-762, miR-1538, miR-1268a, miR-1268b, miR-5095, miR-5096, miR-6727-5p, miR-619-5p, miR-1273 h-5p	No statement about miRNA expression
Plachel et al. [41]	Serum in RCT vs healthy and RCT vs tendinopathy: miR-19b-3p, miR-192-5p, miR-25-3p, miR-19a-3p, miR-18b-5p, miR-93-5p Serum in tendinopathy/RCT vs. healthy: miR-30a-5p, miR-324-3p, miR-210-3p, miR-140-3p, miR-425-5p, miR-222-3p Serum RCT vs. healthy: miR-29a-3p, miR-29c-3p Biopsy: miR-29a-3p, miR-29c-3p, miR-30a-5p, miR-140-3p, miR-192-5p	All miRNAs were significantly downregulated
Xiao et al. [57]	miR-29a	Increase in miR-29a mimics leads to significant down-regulation of BMP2 and BMP12
Thankam et al. [55]	miR-145-5p, miR-99a-5p, miR-100-5p, miR-150-5p, miR-193b-3p, miR-103a-3p, miR-31-5p, miR-195-5p, miR-497-5p, miR-15a-5p, miR-16-5p, let-7b-5p, miR-297	All miRNAs significantly downregulated except miR-297, which was significantly upregulated
Hall et al. [43]	Tendinopathic tendons: miR-199b-5p, miR-26a-5p, miR-532-5p, miR-199a-5p, miR-29a-3p, miR-92a-3p, miR-22-3p, miR-191-5p, miR-10a-5p, miR-199b-3p, miR-199a-3p, miR-126-3p, let-7i-5p, miR-30c-5p, let-7 g-5p, miR-30d-5p, miR-151a-3p Healthy tendons: miR-140-5p, miR-222-3p, let-7e-5p, miR-100-5p,	All miRNAs were significantly downregulated
Ge et al. [48]	miR-148a-3p	miR-148a-3p significantly upregulated in tendinopathy
Thankam et al. [40]	235 miRNAs, of which the 10 most down-regulated were: miR-191-5p, miR-361-5p, miR-1273 g-3p, miR-99b-5p, miR-145-5p, miR-99a-5p, miR-100-5p, miR-23b-3p, miR-425-5p, miR-151a-3p The 10 strongest upregulated: miR-5001-5p, miR-8071, miR-6723-5p, miR-4467, miR-6870-5p, miR-7150, miR-6124, miR-297, miR-4668-5p, miR-8075	Here: also let-7b-5p as well as the miR-25-3p, miR-29a-3p, miR-532-5p, miR-199a-5p and the miR-140-3p significantly downregulated
Han et al. [49]	miR-217	miR-217 significantly increased in old tendons
Leal et al. [44]	miR-29a-3p, miR-29b-3p, miR-29a-5p	Significant inverse correlation with MMPs. No statement about miRNA expression
Brown et al. [46]	miR-608	If MIR608 gene present, significantly lower risk of suffering an Achilles tendon injury
Lu et al. [50]	miR-29b-3p	Increase in miR-29b-3p resulted in a negative effect on tenogenic differentiation
Thankam et al. [54]	miR-125a-5p, miR-145-5p, miR-151a-3p, miR-139-5p, miR-24-3p, miR-130a-3p, miR-155-5p, miR-21-5p, miR-29a-3p, miR-498, miR-132-3p, miR-221-3p, miR-130b-3p, miR-25-3p, miR-337-5p, let-7b-5p, miR-382-5p, miR-199a-5p, miR-140-3p, miR-532-5p, miR-122-5p	All miRNAs, except for miRNA-498, were significantly downregulated
Wang et al. [56]	miR-124	Increase in miR-124 has a negative effect on tendon healing
Hu et al. [60]	miR-138	Increase in miR-138 significantly decreases osteogenic differentiation
Chen et al. [59]	miR-140-5p	Overexpression of miR-140-5p results in significantly reduced PIN1- expression
Millar et al. [51]	miR-29a	Increase in miR-29a resulted in a significant decrease in collagen-3 production
Cai et al. [58]	miR-499, miR-200B, miR-200C, miR-429, miR-149, miR-507, miR-144, miR-502, miR-519C, miR-519B, miR-519A, miR-150, miR-520G, miR-520H, miR-21	No statement about miRNA expression
Peffers et al. [52]	miR-1245A	Significant down-regulation in old tendons compared to young tendons
Poulsen et al. [61]	miR-28-5p	Increase in miR-28-5p leads to apoptosis of tenocytes
Abrahams et al. [45]	miR-608	No statement about miRNA expression

*miR* micro-RNA*, miRNA* microRNA*, hTDSCs* human tendon stem cells*, BMP* Bone morphogenetic protein*, MMPs* matrix metalloproteinases, *PIN1* Peptidyl-prolyl isomerase

### Synthesis of results of miRNAs

Since only studies with, at least in part, a borderline level of evidence were considered for validity purposes, a total of 18 studies were included in the best-evidence synthesis [40–57]. Table 6 shows the corresponding results of miRNAs and their expression patterns associated with tendinopathies and degenerative tendon ruptures. A total of 18 different miRNAs were found that could be detected for more than two times. An evidence level for 12 different miRNAs could be related. Particularly, moderate evidence was found for four miRNAs (miR-25-3p, miR-29a-3p, miR-140-3p, miR-425-5p) and limited evidence for eight miRNAs (miR-99a-5p, miR-145-5p, miR-151a-3p, miR-191-5p, miR-199a-5p, miR-297, miR-532-5p, let-7b-5p). For four miRNAs that appeared multiple times, no evidence (miR-29a, miR-29b-3p miR-608, miR-1273 g-3p) could be identified, because the regulatory pattern was unclear. For two miRNAs (miR-100-5p and miR-222-3p), the results were conflicting.

**Table 6:** Best-evidence-synthesis of miRNAs associated with tendinopathies or tendon ruptures

Study	miRNA	Expression pattern	Sample		Study quality	Overall Rating
Plachel et al. [41] Thankam et al. [40] Thankam et al. [54]	25-3p	↓ ↓ ↓	Venous blood Bicep tendon Bicep tendon		High Quality Borderline Borderline	Moderate evidence
Plachel et al. [41] Hall et al. [43] Thankam et al. [40] Leal et al. [44] Thankam et al. [54]	29a-3p	↓ ↓ ↓ → ↓	Venous blood, SSP/SSC tendon SSP/SSC tendon Bicep tendon SSP tendon Bicep tendon		High Quality Acceptable Borderline Acceptable Borderline	Moderate evidence
Plachel et al. [41] Thankam et al. [40] Thankam et al. [54]	140-3p	↓ ↓ ↓	Venous blood, SSP/SSC tendon Bicep tendon Bicep tendon		High Quality Borderline Borderline	Moderate evidence
Plachel et al. [41] Thankam et al. [40]	425-5p	↓ ↓	Venous blood Bicep tendon		High Quality Borderline	Moderate evidence
Thankam et al. [55] Thankam et al. [40]	99a-5p	↓ ↓	Bicep tendon Bicep tendon		Borderline Borderline	Limited evidence
Thankam et al. [55] Thankam et al. [40] Thankam et al. [54]	145-5p	↓ ↓ ↓	Bicep tendon Bicep tendon Bicep tendon		Borderline Borderline Borderline	Limited evidence
Hall et al. [43] Thankam et al. [40] Thankam et al. [54]	151a-3p	↓ ↓ ↓	SSP/SSC tendon Bicep tendon Bicep tendon		Acceptable Borderline Borderline	Limited evidence
Hall et al. [43] Thankam et al. [40]	191-5p	↓ ↓	SSP/SSC tendon Bicep tendon		Acceptable Borderline	Limited evidence
Hall et al. [43] Thankam et al. [40] Thankam et al. [54]	199a-5p	↓ ↓ ↓	SSP/SSC tendon Bicep tendon Bicep tendon	Acceptable Borderline Borderline		Limited evidence
Thankam et al. [55] Thankam et al. [40]	297	↑ ↑	Bicep tendon Bicep tendon	Borderline Borderline		Limited evidence
Hall et al. [43] Thankam et al. [40] Thankam et al. [54]	532-5p	↓ ↓ ↓	SSP/SSC tendon Bicep tendon Bicep tendon	Acceptable Borderline Borderline		Limited evidence
Thankam et al. [55] Thankam et al. [40] Thankam et al. [54]	let-7b-5p	↓ ↓ ↓	Bicep tendon Bicep tendon Bicep tendon	Borderline Borderline Borderline		Limited evidence
Thankam et al. [55] Hall et al. [43] Thankam et al. [40]	100-5p	↓ → ↓	Bicep tendon SSP/SSC tendon Bicep tendon	Borderline Acceptable Borderline		Conflicting evidence
Plachel et al. [41] Hall et al. [43]	222-3p	↓ →	Venous blood SSP/SSC tendon	High quality Acceptable		Conflicting evidence
Xiao et al. [57] Millar et al. [51]	29a	→ →	Patellar tendon SSP/SSC tendon	Borderline Borderline		No evidence
Leal et al. [44] Lu et al. [50]	29b-3p	→ →	SSP tendon Hamstring tendon	Acceptable Borderline		No evidence
Brown et al. [46] Abrahams et al. [45]	608	→ →	Saliva Unclear	Borderline Borderline		No evidence
Ge et al. [42] Thankam et al. [40]	1273 g-3p	→ ↓	SSP tendon Bicep tendon	Acceptable Borderline		No evidence

↑ upregulated, ↓ downregulated, → neutral, *SSP* Supraspinatus, *SSC* Subscapularis, *miRNA* microRNA

## Discussion

The main finding was that moderate evidence was found for miR-140-3p and miR-425-5p as potential biomarkers for tendinopathies as well as for miR-25-3p, miR-29-a-3p, miR-140-3p, and miR-425-5p for the detection of degenerative tendon ruptures. This evidence applies to tendons at the upper extremity in elderly patients. All miRNAs were associated with inflammatory cytokines as interleukin-6(ß) and tumor necrosis factor alpha.

Moderate evidence exists for miR-25-3p, miR-29a-3p, miR-140-3p, and miR-425–5 as potential biomarkers for pathological tendon conditions (Table 6). Our findings are in line with those of previous systematic reviews [31, 32], showing that the miR-29 family have a special importance in such tendon diseases. Our review adds that moderate evidence was found for miR-25-3p, miR-29a-3p, miR-140-3p, and miR-425-5p as biomarkers for pathological tendon conditions (Table 6); exclusively, at the upper extremity associated either with biceps [40, 54], supraspinatus/subscapularis [41, 43], or supraspinatus [44] tendons in elderly patients. Furthermore, significant differences in the circulation were found for both miR-140-3p and miR-425-5p for tendinopathic tendons compared with healthy tendons [41] and for miR-25-3p, miR-29a-3p, miR-140-3p, and miR-425-5p in degenerative tendon ruptures compared with healthy tendons [41]. However, the sampling was exclusively taken in elderly [41, 43, 44] or patients with unknown age [40, 54]. Thus, there is not only a need for more high-quality studies, but also for more potential miRNAs in tendon diseases at the lower extremity and in patients at younger ages.

MiR-140-3p and miR-425-5p could serve as potential biomarkers for tendinopathies (Table 6). For both miRNAs, significantly decreased expression levels were observed in tendinopathic tendons in the circulation, when compared to healthy tendons [41]. In addition, the study by Thankam et al. [40] found that both miRNAs were significantly decreased in tendon injuries with glenohumeral arthritis compared to healthy control tendons. However, it is important to emphasize that these were not tendinopathic, but tendons with massive tears. Moreover, miR-140-3p was significantly decreased in tendinopathic tendons with glenohumeral arthritis compared to tendinopathic tendons without glenohumeral arthritis [54]. Thus, miR-140-3p and miR-425-5p may be potential diagnostic biomarkers for tendinopathies, but the results should be taken with caution due to the association found with further diseases.

MiR-25-3p, miR-29-a-3p, miR-140-3p, and miR-425-5p could serve as potential biomarkers for the detection of degenerative tendon ruptures (Table 6) due to the significant downregulation in the circulation in degenerative ruptured tendons compared with healthy tendons [41]. Here, miR-29a-3p and miR140-3p were shown to be significantly downregulated in both tissue and circulation in degenerative ruptured tendons [41]. Additionally, the study by Thankam et al. [40] demonstrated that miR-25-3p, miR-29a-3p, miR-140-3p, and miR-425-5p were also significantly downregulated in tendon ruptures of the bicep tendon compared with healthy control tendons. In the study by Thankam et al. [54], it was shown that miR-25-3p, miR-29a-3p, and miR-140-3p were also significantly downregulated in tendinopathic tendons with glenohumeral arthritis compared with tendinopathic biceps tendons. Furthermore, miR-29a-3p was downregulated in tissue in tendinopathic supraspinatus tendons compared with healthy subscapularis tendons [43]. In a study by Leal et al. [44], miR-29a-3p was inversely correlated with various matrix metalloproteinases (MMPs), but there were no significant differences in the expression rates of miR-29a-3p between healthy and ruptured supraspinatus tendons. Thus, there seems to be a relationship between miR-25-3p, miR-29a-3p, miR-140-3p, and miR-425-5p with degenerative tendon ruptures. MiR-140-3p and miR-425-5p were significantly downregulated in both tendinopathic tendons and degenerative tendons in the circulation compared with healthy control tendons. A progressive decrease in expression levels was also observed for the two miRNAs in relation to the severity of tendon degeneration [41]. This suggests that miR-140-3p and miR-425-5p may contribute to the pathogenesis and/or progression of degenerative rotator cuff diseases in elderly patients, requiring further validation.

Different regulatory mechanisms of miRNAs in tendon tissue are discussed in the literature. Briefly, miR-25-3p can be considered as a potential tumor biomarker in breast cancer [62] or osteosarcoma [63]. In both cases, cytokines such as interleukin-6 (Il-6) influence tumor genesis [64, 65] and Il-6 also plays a role in tendon ruptures [66]. For miR-29a-3p, it has been shown to be an eligible biomarker in colorectal cancer [21] and tuberculosis [67], among others. In both tuberculosis and carciogenesis, Il-6 play an important role again [68, 69]. Regarding the miR-140-3p, it is evident that this miRNA is also significantly down-regulated in human chondrocytes in glenohumeral arthritis [70], among others. MiR-140-3p was shown to reduce the concentration of interleukin-1ß (IL-1ß) induced inflammatory factors [70]. IL1-ß plays a crucial role mainly in the inflammatory phase of tendon healing [71], but it has also been shown that it has a significant role in arthritis [72]. Gu et al. [73] demonstrated that miR-425-5p is associated with both tumor necrosis factor alpha (TNF-alpha) and IL-1ß, which also plays a role in tendinopathies [71]. Overall, all miRNAs for which moderate evidence was found are associated with specific inflammatory cytokines. Therefore, it is unclear, if these miRNAs can serve as potential biomarkers for tendon diseases or significantly alter their expression patterns tissue-independently due to inflammatory processes. More experimental high-quality research is needed to validate miR-25-3p, miR-29a-3p, miR-140-3p, and miR-425-5p as tendon-specific biomarkers.

Although this systematic review increased the knowledge on miRNAs as potential biomarkers for tendon diseases, there are few limitations. While Pubmed can be regarded as the most comprehensive database, it has to be noted that it was the only platform used for the literature search. Additionally, all methodological steps of our review were conducted only by one author. However, a second author carefully validated the entire proceed and all outcomes independently, which is not fully compliant with the PRISMA guidelines. More experimental high-quality studies are needed to investigate miRNAs in both the tissue and circulation to validate them as biomarkers for tendinopathies or degenerative tendon ruptures; especially, at the lower extremity and in younger individuals. Also, more basic research is required to better understand the regulatory mechanisms of miRNAs within the tendon pathophysiology.

## Conclusion

Our systematic review based on a best-evidence synthesis suggests that moderate evidence exists for four miRNAs as potential biomarkers for tendinopathies and degenerative tendon ruptures at the upper extremity in elderly patients. The identified miRNAs are associated with inflammatory processes. More experimental high-quality research to validate the four miRNAs is required.

## Data Availability

The datasets used and analysed during the current study are available from the corresponding author on reasonable request.

## Abbreviations

IL-1ß: Interleukin-1ß
IL-6: Interleukin-6
PRISMA: Preferred Reporting Items for Systematic Reviews and Meta-analyses
ROM: Range of motion
SIGN: Scottish Intercollegiate Guidelines Network
TGF-ß1: Transforming growth factor ß1
TNF-alpha: Tumor necrosis factor alpha
